# Blue light regenerates functional visual pigments in mammals through a retinyl-phospholipid intermediate

**DOI:** 10.1038/s41467-017-00018-4

**Published:** 2017-05-04

**Authors:** Joanna J. Kaylor, Tongzhou Xu, Norianne T. Ingram, Avian Tsan, Hayk Hakobyan, Gordon L. Fain, Gabriel H. Travis

**Affiliations:** 1 Jules Stein Eye Institute, University of California Los Angeles School of Medicine, Los Angeles, California 90095 USA; 2 Molecular, Cellular and Integrative Physiology Graduate Program, University of California Los Angeles School of Medicine, Los Angeles, California 90095 USA; 3 Department of Integrative Biology and Physiology, University of California Los Angeles School of Medicine, Los Angeles, California 90095 USA; 4 Department of Biological Chemistry, University of California Los Angeles School of Medicine, Los Angeles, California 90095 USA

## Abstract

The light absorbing chromophore in opsin visual pigments is the protonated Schiff base of 11-*cis-*retinaldehyde (11cRAL). Absorption of a photon isomerizes 11cRAL to all-*trans-*retinaldehyde (atRAL), briefly activating the pigment before it dissociates. Light sensitivity is restored when apo-opsin combines with another 11cRAL to form a new visual pigment. Conversion of atRAL to 11cRAL is carried out by enzyme pathways in neighboring cells. Here we show that blue (450-nm) light converts atRAL specifically to 11cRAL through a retinyl-phospholipid intermediate in photoreceptor membranes. The quantum efficiency of this photoconversion is similar to rhodopsin. Photoreceptor membranes synthesize 11cRAL chromophore faster under blue light than in darkness. Live mice regenerate rhodopsin more rapidly in blue light. Finally, whole retinas and isolated cone cells show increased photosensitivity following exposure to blue light. These results indicate that light contributes to visual-pigment renewal in mammalian rods and cones through a non-enzymatic process involving retinyl-phospholipids.

## Abstract (web summary)

It is currently thought that visual pigments in vertebrate photoreceptors are regenerated exclusively through enzymatic cycles. Here the authors show that mammalian photoreceptors also regenerate opsin pigments in light through photoisomerization of *N*-ret-PE (*N*-retinylidene-phosphatidylethanolamine.

## Introduction

Light perception in metazoans is mediated by two types of photosensitive cells, rhabdomeric and ciliary photoreceptors. Both contain membranous structures filled with opsin pigments. Ciliary photoreceptors, such as human rods and cones, contain an outer segment (OS) comprising a stack of membranous disks. The first event in light perception is capture of a photon by an opsin pigment. The light-absorbing chromophore in most opsins is 11-*cis*-retinaldehyde (11cRAL) coupled to a lysine residue through a protonated Schiff-base linkage. Absorption of a photon isomerizes the 11cRAL to all*-trans*-retinaldehyde (atRAL), transiently converting the pigment to its active (metarhodopsin II/III) signaling state. In the rhabdomeric photoreceptors of insects, atRAL remains covalently coupled to the opsin following activation. Absorption of a second photon flips the atRAL back to 11cRAL, restoring light sensitivity through photoregeneration^1^. For this reason, rhabdomeric opsins are called bistable pigments. In bright light they flicker between signaling and light-sensitive forms.

The opsin pigments of ciliary photoreceptors decay following photoactivation to yield unliganded apo-opsin and free atRAL^2^. Ciliary opsins are hence called bleaching pigments. Immediately following photon absorption by a ciliary opsin, the resulting metarhodopsin I may absorb a second photon, converting the atRAL back to 11cRAL, and the pigment to its light sensitive state^3, 4^. Thus, during the first millisecond of the rhodopsin cycle, ciliary rhodopsin behaves as a bistable pigment. After deprotonation of the Schiff base with activation of the pigment, photoreversal no longer occurs^5, 6^. Photoreversal therefore contributes negligibly to pigment regeneration in ciliary photoreceptors. Light sensitivity is restored to apo-opsin when it combines with another 11cRAL to form rhodopsin. The conversion of atRAL back to 11cRAL is carried out by multi-step enzyme pathways in cells of the retinal pigment epithelium (RPE)^7^ and Müller glial cells in the retina^8, 9, 10^. Thus, rods and cones rely on enzymatic reactions in neighboring cells to synthesize visual chromophore, and appear not to benefit from the faster photoregeneration employed by “lower” metazoan species. For sustained vision in daylight, ciliary photoreceptors must be supplied with fresh 11cRAL at a rate that matches the rate of chromophore consumption through photoisomerization of opsins.

Retinaldehydes are lipophilic with low aqueous solubility^11^. They are present at high concentrations in OS disk membranes, which serve as conduits for 11cRAL and atRAL flowing to and from the opsins. Opsin crystal structures show openings to the ligand-binding cavity between pairs of transmembrane helices^12, 13^. Retinaldehydes are thought to enter and exit the chromophore-binding site of opsin via these openings. In the disk bilayer, retinaldehydes rapidly and reversibly condense with phosphatidylethanolamine (PE) to form the retinyl-lipid, *N-*retinylidene-PE (*N-*ret-PE)^14, 15^. Importantly, it was previously shown that all-*trans-* (at-) *N-*ret-PE undergoes photoisomerization to 11-*cis-* (11c-) *N-*ret-PE in visible light^16^, and that 11c-*N-*ret-PE transfers 11cRAL to apo-opsin^14, 17^. Photoregeneration of visual pigments has never been reported in vertebrates. It is currently thought that visual pigments in vertebrate photoreceptors are regenerated exclusively by the enzymatic visual cycles. Here, we show that mammalian photoreceptors possess a mechanism for light-driven regeneration of opsin pigments, employing *N-*ret-PE as a light-sensitive intermediate. This mechanism is distinct from both photoregeneration of bistable opsins in rhabdomeric photoreceptors and the enzymatic visual cycles in RPE and Müller cells of vertebrates.

## Results

### Photoisomerization of *N-*ret-PE

We synthesized at-*N-*ret-PE and determined its absorbance spectra in acidified or alkalized methanol. The maximum absorption wavelength (*λ*_max_) of protonated at-*N-*ret-PE was 450 nm, while the *λ*_max_ of non-protonated at-*N-*ret-PE was 365 nm (Supplementary Fig. 1). The p*K*_a_ of *N-*ret-PE is 6.9^18^. Since the pH near the surface of biological membranes is approximately one pH-unit lower than the surrounding aqueous medium^19^, most *N-*ret-PE is protonated in vivo. Non-protonated *N-*ret-PE probably contributes little to chromophore photoregeneration because of its low abundance, and because the optic media blocks transmission of light below 400 nm^20^.

**Fig. 1:**
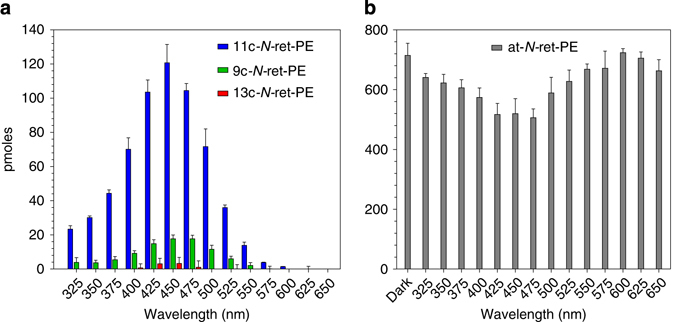
Action spectrum for photoisomerization of protonated at-*N-*ret-PE. Protonated at-*N-*ret-PE in acidified methanol was incubated in the dark or under monochromatic light of the indicated wavelengths and the same photon flux. **a** Molar composition of 11c-, 9c- and 13c-*N-*ret-PE isomers following the incubation in light. The dark background level of each isomer was subtracted. Note the 37-fold higher level of 11c- versus 13c-*N-*ret-PE in the 450-nm samples. **b** Molar amounts of at-*N-*ret-PE remaining after incubation in the dark or in light of the indicated wavelengths. Data are plotted as mean ± s.d. (*n* = 3)

We tested whether *N-*ret-PE undergoes at-to-11c photoisomerization in light, as previously observed^16^. To this end, we exposed samples of protonated at-*N-*ret-PE to monochromatic light of wavelengths 325–650 nm for 80 s, each with a photon flux of 0.95 µmol photons/m^2^ s. We determined the isomer composition of *N-*ret-PE for each wavelength by reacting the samples with hydroxylamine to form stable retinaldehyde oximes and quantitating by normal-phase liquid chromatography (LC). We observed dramatic light-dependent conversion of at-*N-*ret-PE to its 11c-isomer (Fig. 1). The action spectrum for synthesis of 11c-*N-*ret-PE was nearly identical to the UV-visible absorbance spectrum of protonated at-*N-*ret-PE, both exhibiting maxima at ~450 nm (Fig. 1 and Supplementary Fig. 1). Light-dependent synthesis of the 9c-isomer and 13c-isomer also exhibited maxima at 450 nm, but were formed in much lower amounts than the 11c-isomer (Fig. 1a). Consistently, at-*N-*ret-PE showed light-dependent depletion, with an inverted action spectrum, also centered at 450 nm (Fig. 1b). To quantitate light-dependent formation of *cis*-isomers, we subtracted the amount of each *cis*-isomer in the dark-incubated samples from that in the 450-nm light-exposed samples (Fig. 1a). This yielded 121 pmol of 11c-, 18 pmol of 9-*cis-* (9c-), and 3.3 pmol of 13-*cis-* (13c-) *N-*ret-PE, balanced by 195 pmol of at-*N-*ret-PE consumed. These pmole values have relative but not absolute meaning. The ratio of 11cRAL to 13cRAL following photoisomerization of *N-*ret-PE was 37:1. Efficient photoconversion of at- to 11c-*N-*ret-PE suggests that *N-*ret-PE in OS may be a source of chromophore for the visual opsins in light-exposed retinas.

### Synthesis of rhodopsin by OS membranes exposed to blue light

**Fig. 2:**
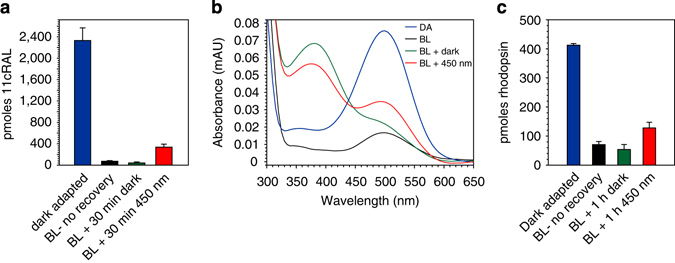
Light-dependent regeneration of 11cRAL and rhodopsin in bovine OS. Aliquots of rod OS from dark-adapted bovine retinas were analyzed before and after a deep photobleach. After addition of atRAL, the remaining aliquots were incubated in the dark or in 450-nm light at 0.5 W/m^2^ for the indicated times. **a** Levels of 11cRAL in dark-adapted (DA), immediate post-bleach (BL—no recovery), post-bleach plus 30 min incubation with atRAL in the dark (BL + 30 min dark), or post-bleach plus 30 min incubation with atRAL in 450-nm light (BL + 30 min 450 nm) OS. Data are plotted as mean ± s.d. (*n* = 3). **b** Representative UV-visible spectra of affinity-purified rhodopsin from bovine OS treated as described in panel **c**. The prominent 360-370-nm shoulders in the rhodopsin spectra from the (BL + 1 h dark) and (BL + 1 h. 450 nm) samples represent the added atRAL. **c** Levels of rhodopsin in bovine OS from dark-adapted bovine eyes (DA), immediately following a photobleach (BL - no recovery), incubated for 1 h in the dark with atRAL following the photobleach (BL + 1 h dark), or incubated for 1 h in 450-nm light with atRAL following the photobleach (BL + 1 h 450 nm). Data are plotted as mean ± s.d. (*n* = 3)

Next, we tested whether 450-nm light could induce synthesis of 11cRAL in native OS membranes. We prepared rod OS from the retinas of fresh, ex vivo dark-adapted bovine eyes. Equal aliquots of fresh OS membranes were exposed to UV-filtered white light (400-nm cutoff) to bleach the rhodopsin. Following addition of atRAL, one set of OS samples was placed in the dark while a second set was exposed to 450-nm monochromatic light for 30 min. The OS samples were extracted and analyzed for retinoid content by normal-phase LC. The UV-filtered white light photobleached approximately 95% of 11cRAL in the OS samples, indicating that most was in the form of rhodopsin since it was sensitive to visible light (Fig. 2a). OS samples exposed to 450-nm light for 30 min yielded a 7.8-fold increase in total 11cRAL over samples kept in the dark (Fig. 2a). These data suggest that OS membranes support light-driven synthesis of visual chromophore.

To test for light-dependent synthesis of rhodopsin, we again prepared fresh, dark-adapted bovine OS membranes. We divided the OS into samples containing two nmoles of rhodopsin. Some were photobleached by exposure to UV-filtered white light for 45 min while the remaining samples were kept in the dark. Ten nmoles of atRAL substrate was added to the photobleached samples. One set was incubated for 1 h in the dark while the other set was exposed to 450-nm light. We purified opsin protein from the OS samples by immunoaffinity chromatography and quantitated rhodopsin pigment by UV-visible absorbance spectroscopy (Figs 2b and c). The initial exposure to white light bleached 83% of the rhodopsin. No recovery of rhodopsin was observed following 1-h incubation in the dark. This was expected since the required chromophore-regenerating enzymes are not present in OS. In contrast, rhodopsin increased 2.4-fold in OS samples exposed to 450-nm light (Fig. 2c). These data show that free atRAL efficiently combines with PE to form at-*N-*ret-PE, and that following photoisomerization, 11c-*N-*ret-PE efficiently donates 11cRAL to regenerate rhodopsin in OS membranes.

### *N-*ret-PE in dark-adapted mouse retinas

Here we measured *N-*ret-PE in retinas from dark-adapted wild type (strain 129/Sv) mice. We extracted phospholipids from retina homogenates and separated them by reverse-phase LC. Three doublet peaks, all with *λ*_max_ near 450 nm, eluted between 21 and 38 min (Supplementary Fig. 2a). These peaks likely represent different fatty-acyl forms of *N-*ret-PE. We collected the eluates corresponding to these peaks, reacted the pooled fractions with hydroxylamine to form retinaldehyde oximes, and separated the oximes by normal-phase LC (Supplementary Fig. 2b). This allowed us to quantitate the retinaldehyde isomers of *N-*ret-PE in dark-adapted mouse retinas (Table 1). We also quantitated total retinaldehydes in dark-adapted (DA) 129/Sv mouse retinas (Table 1). Total retinaldehydes, representing mainly rhodopsin, contained predominantly 11cRAL, while *N-*ret-PE contained higher fractions of 13cRAL and atRAL (Table 1) reflecting the much lower thermal stability of *N-*ret-PE versus rhodopsin^21^. By combining the retinaldehyde isomers in *N-*ret-PE, we quantitated total *N-*ret-PE per dark-adapted mouse retina as 37 pmol (Table 1), or approximately 7% of the total retinaldehyde pool.

**Table 1:** Retinaldehydes in *N-*ret-PE and total retinaldehydes in DA mouse retinas (pmoles per retina)

	11cRAL	atRAL	13cRAL	9cRAL	Combined RALs
Retinaldehydes in *N-*ret-PE	10.0 ± 0.7	18.1 ± 1.3	8.0 ± 0.6	0.9 ± 0.1	37.0 ± 2.7
Total retinaldehydes	454.0 ± 25	72.3 ± 6.1	22.1 ± 3.6	7.9 ± 1.4	556.3 ± 35

### Quantum efficiency of *N-*Ret-PE

**Fig. 3:**
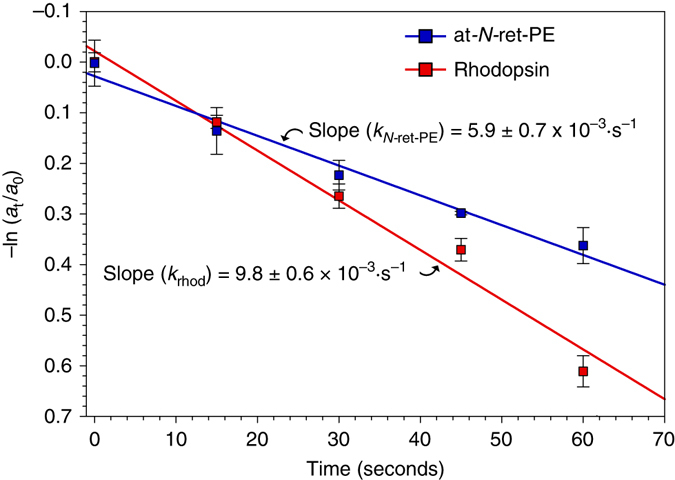
Kinetics of rhodopsin and *N-*ret-PE photoisomerization. Protonated at-*N-*ret-PE and rhodopsin were exposed to 450-nm or 500-nm monochromatic light respectively at the same photon flux for the indicated times. The initial amounts of at-*N-*ret-PE and rhodopsin are represented by *a*_0_, and the amounts at time *t* by *a*_t_. The first-order rate constant (*k*) is described by: *kt* =  −In(*a*_t_/*a*_0_). Here, the slopes of the plots −In(*a*_t_/*a*_0_) versus *t* yield the rate constants *k*_(*N-*ret-PE)_ and *k*_(rhod)_. Each data point is shown as the mean ± s.e.m. (*n* = 3). The rate constants are also shown as mean ± s.e.m

Upon exposure to 450-nm light, at-*N-*ret-PE is specifically converted to 11c-*N-*ret-PE (Fig. 1). Accordingly, we compared the rates of protonated at-*N-*ret-PE disappearance in 450-nm light to rhodopsin disappearance in 500-nm light, both with photon fluxes of 1.0 µmol/m^2^ s. We plotted –ln(*a*_t_ / *a*_0_) versus time, where *a*_t_ = amount of at-*N-*ret-PE or rhodopsin after illumination for *t* seconds and *a*_*0*_ = initial amount without illumination (*t* = 0). This plot yielded the first-order rate constants, *k*_*N-*ret-PE_ and *k*_rhod_ (Fig. 3). We determined the quantum efficiency^22^ for *N-*ret-PE (Φ_*N*-ret-PE_) by the relationship shown in Eq. 1, as previously described^23, 24^.

1 $${{\Phi }}_{N-\rm ret-PE}=\frac{{{\varepsilon }}_{\rm rhod}}{{{\varepsilon }}_{N-\rm ret-PE}}\times \frac{{k}_{N-\rm ret-PE}}{{k}_{\rm rhod}}\times {{\Phi }}_{\rm rhod}$$

By inserting the molar extinction coefficients (*Ɛ*) for rhodopsin at 500 nm (40,600 M¯^1^ cm¯^1^)^25^ and *N-*ret-PE at 450 nm (31,300 M^−1^ cm^−1^),^15^ and the published quantum efficiency for rhodopsin (Φ_rhod_ *= *0.65),^26^ we determined that Φ_*N*-ret-PE_ = 0.51 ± 0.07 (mean ± s.e.m.). The quantum efficiencies of rhodopsin and protonated *N-*ret-PE are therefore similar. The quantum efficiencies of cone opsins are similar to that of rhodopsin^27^ and hence also to *N-*ret-PE.

### Light-stimulated synthesis of chromophore by mouse retinas

Retinal G protein coupled receptor (RGR) opsin is a non-visual opsin in RPE and Müller cells of the retina^28^. Based on its similarity to squid retinochrome, and the phenotype of delayed rhodopsin regeneration in *Rgr* ^−/−^ mutant mice, RGR-opsin was proposed to function as a “reverse” photoisomerase for synthesis of visual chromophore^29^. The *λ*_max_ of protonated RGR-opsin is 469 nm^30^, close to the *λ*_max_ of protonated *N-*ret-PE. Here we tested whether RGR-opsin contributes to the observed 450-nm light-dependent synthesis of 11cRAL. We prepared retina homogenates from wild type (strain 129/Sv) and *Rgr* ^−/−^ mutant (strain 129/Sv background) mice. After photobleaching the homogenates in UV-filtered (400 nm cutoff) white light, we added all-*trans-*retinol (atROL) substrate and incubated the homogenates in the dark or under 450-nm monochromatic light. Retinaldehydes formed during these incubations were quantitated by normal phase LC. As with bovine OS membranes (Fig. 2), the concentration of 11cRAL was approximately eight-fold higher in wild-type mouse retina homogenates exposed to 450-nm light compared to homogenates kept in the dark (Supplementary Fig. 3a). We observed no light-dependent stimulation of 9cRAL or 13cRAL (Supplementary Figs 3b,c), and the expected light dependent consumption of atRAL (Supplementary Fig. 3d). The high levels of 9cRAL and 13cRAL versus 11cRAL in retinal homogenates kept in the dark (Supplementary Figs 3b–d) is due to thermal isomerization of atRAL during the incubations^9^. Loss of RGR-opsin in *Rgr* ^−/−^ mouse retina homogenates had no effect on light-dependent formation of 11cRAL, in fact levels of 11cRAL were marginally higher in *Rgr* ^−/−^ versus wild-type retina homogenates exposed to 450-nm light (Supplementary Fig. 3a). As with wild-type mouse retinas, levels of 9cRAL and 13cRAL were not increased in *Rgr* ^−/−^ retinas following exposure to 450-nm light (Supplementary Figs 3b,c), while levels of atRAL exhibited a similar compensatory reduction (Supplementary Fig. 3d). These data suggest that RGR-opsin is not responsible for the observed blue light-dependent synthesis of visual chromophore.

### Accelerated recovery of rhodopsin in live mice by blue light

**Fig. 4:**
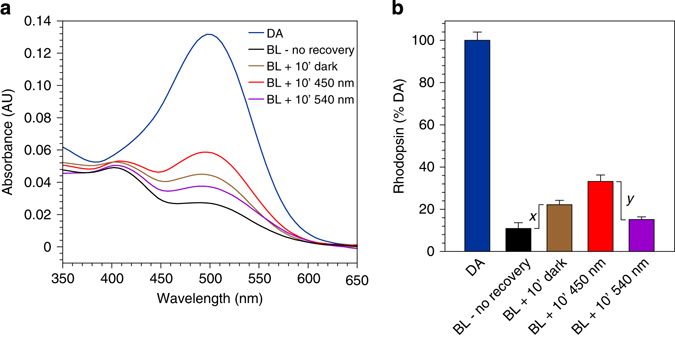
Light dependent regeneration of rhodopsin in live mice. Wild-type mice were dark adapted overnight and exposed to the following light conditions: dark-adapted (DA); 90% strobe bleach with no recovery time (BL – no recovery); strobe bleach with 10 min recovery in the dark (BL + 10’ dark); strobe bleach with 10 min recovery in 450-nm light (BL + 10’ 450 nm); or strobe bleach with 10 min recovery in 540-nm light (BL + 540 nm). The photon fluxes of the 450-nm and 540-nm light were identical. The mice were euthanized, their retinas collected, and affinity purification of rhodopsin was carried out on the retina homogenates. **a** Representative baseline-normalized UV-Vis spectra of purified rhodopsin from mice exposed to the indicated light conditions. **b** Levels of rhodopsin visual pigment in retinas from mice exposed to the indicated light conditions, expressed as percent of dark-adapted rhodopsin. The value *x* represents the increase in rhodopsin during post-bleach recovery. The value *y* represents the difference in rhodopsin between mice exposed to 450-nm versus 540-nm light during recovery. Error bars show ± s.d. (n = 3)

To determine whether blue-light stimulates rhodopsin regeneration in vivo, we dark-adapted wild type (129/Sv) mice overnight. The mice were anesthetized and exposed to UV-filtered strobe light to bleach approximately 90% of the rhodopsin. We allowed one group of mice to recover in darkness, exposed another group to 450-nm light, and exposed the third group to 540-nm light, all for 10 min. Photon fluxes were the same for the 450-nm and 540-nm light (1.8 µmol/m^2^ s). We chose 540 nm to complement 450 nm because these wavelengths bracket the *λ*_max_ of rhodopsin (500 nm) and are equally efficient at photoisomerizing rhodopsin. Immediately after the recovery period, we euthanized the mice, collected and homogenized their retinas, and performed immunoaffinity separation to isolate rhodopsin protein. To confirm similar recoveries of affinity purified rhodopsin protein independent of its ligand state, we determined the protein concentration of each supernatant fraction. The concentrations were similar, with a global average of 162 ± 18 µg/ml (s.e.m., *n* = 15). Next, we measured the amounts of functional rhodopsin in the same eluates by measuring the difference in 500-nm absorbance before and after bleaching with a strobe (Figs 4a, b).

Functional rhodopsin approximately doubled in samples from mice allowed to recover in the dark for 10 min versus samples from mice collected immediately following the bleach (Fig. 4b). Mice exposed to 450-nm light during post-bleach recovery showed an additional 1.5-fold increase in percent rhodopsin above the amount in mice that recovered in darkness (Fig. 4b). Finally, retinas from mice exposed to 540-nm light during recovery contained a lower percent of rhodopsin than retinas from mice that either recovered in the dark or were exposed to 450-nm light (Fig. 4b). The difference in rhodopsin levels between mice bleached with no recovery period and mice bleached with 10 min recovery in the dark represents rhodopsin regenerated by the enzymatic visual cycles (*x* in Fig. 4b). The difference in rhodopsin levels between mice that recovered in 450-nm light and mice that recovered in 540-nm light represents rhodopsin regenerated through photoisomerization of *N-*ret-PE (*y* in Fig. 4b). This applies because the 450-nm and 540-nm light photoisomerize rhodopsin with similar efficiency (Fig. 4a), while 540-nm light has little effect on protonated *N-*ret-PE (Fig. 1 and Supplementary Fig. 1). The radiant energies used in this experiment were 0.5 W/m^2^ and 0.42 W/m^2^ for the 450-nm and 540-nm light, respectively, yielding identical photon fluxes (1.8 µmol photons/m^2^ s), similar to the radiant energy in a typical office environment. These results establish that blue-light dependent regeneration of rhodopsin occurs in live mice.

### Increased photosensitivity of cones exposed to blue light

**Fig. 5:**
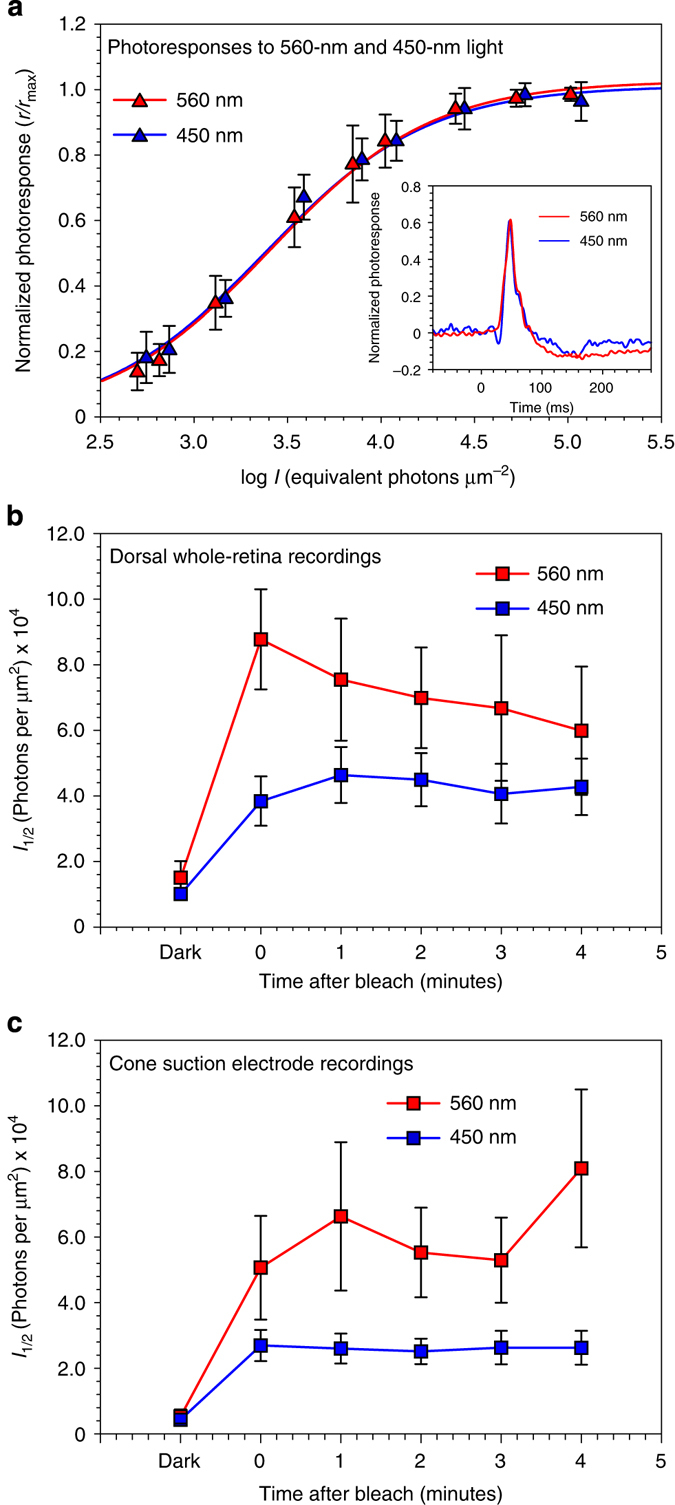
Photosensitivity in *Gnat1* ^−/−^ cones after exposure to 450-nm or 560-nm light. Photoresponses were recorded from M (508-nm) cones in *Gnat1* ^−/−^ retinas before and after 15-second exposures to 450-nm or 560-nm light calculated to bleach 85% of the M-cone pigment. **a** Mean suction-electrode responses (±s.e.m.) from dark-adapted M cones as a function of flash intensity to 450-nm (*n* = 7) and 560-nm (*n* = 10) light at the same LED current and flash durations. Peak response amplitudes (*r*) were separately normalized to maximum peak response amplitudes at saturating light intensities (*r*_max_) for the two wavelengths, and intensities were multiplied by 0.6 to give equivalent intensities at the *λ*_max_ of the M-cone pigment. Curves are Michaelis-Menten equation with values of *I*_1/2_ of 2410 for 450 nm and 2560 for 560 nm (in equivalent photons μm^−2^). Inset: mean responses to 450-nm and 560-nm light of flashes 2.5 ms in duration at intensities of approximately 3600 equivalent photons μm^−2^. **b**,**c** Mean M-cone responses (±s.e.m.) before and after bleaching 85% of the cone visual pigment from **b** whole dorsal *Gnat1* ^−/−^ retinas from five mice and **c** suction-electrode recordings from single *Gnat1* ^−/−^ cones (*n* = 14 for 450-nm bleach, *n* = 12 for 560-nm bleach). Bleaching was performed with the same 450-nm and 560-nm light sources used in **a**. Cones and whole retinas were stimulated before and after the photobleach with three 500-nm flash intensities spanning the range of cone responses from small to nearly saturating. Mean response amplitudes were used to estimate the intensity required to produce a half-maximal response from fits to the Michaelis–Menten equation

Rods comprise > 90% of photoreceptors in retinas from most mammalian species including mice, cattle and humans^31, 32^. Given the small percentage of cones, the blue light-dependent synthesis of 11cRAL observed in bovine OS and mouse retinas (Figs 2, 4) mainly reflects retinyl-lipid photoisomerization in rods. To test if photoisomerization of *N-*ret-PE contributes to regeneration of cone opsins, we made electrical recordings of cone photoresponses before and after bleaching with 450 nm or 560 nm monochromatic light. We chose these two wavelengths because the M-cones are equally sensitive to 450-nm and 560-nm light, while at-*N-*ret-PE is only photoisomerized to 11c-*N-*ret-PE by 450-nm light (Figs 1, 2). To do these experiments, we used a white-light LED and narrow-band interference filters. The absolute intensities at 450 nm and 560 nm (as measured with a calibrated photodiode) were nearly identical at all LED voltages. To demonstrate their equivalence, we measured the response of single M-cones at these two wavelengths as a function of LED intensity (Fig. 5a). Because the maximum amplitude of the current responses varied somewhat from cell to cell, primarily as the result of small differences in the seal of the suction pipette around the cell, we normalized responses (*R*) to maximum response amplitudes (*R*_max_) at each of the two wavelengths. After normalization, response amplitudes as a function of flash intensity were nearly identical.

We then recorded photovoltages from M-cone enriched whole dorsal retinas (Fig. 5b) and suction-electrode responses from single M-cones (Fig. 5c). All of our M-cone recordings (including those in Fig. 5a) were made from *Gnat1* ^−/−^ mice lacking rod α-transducin, which exhibit normal cone photoresponses but no detectable rod response^33^. We exposed retinas or groups of cells to either 450-nm or 560-nm monochromatic light for 15 sec at the same photon fluxes calculated to bleach 85% of cone M opsin^34, 35^, assuming a pigment photosensitivity of 6 × 10^9^ μm^2^. After bleaching with either wavelength, M-cones were stimulated at 500 nm, near the *λ*_max_ of M-cone opsin (508 nm).

The results of these experiments are shown in Fig. 5b for whole-retina recordings and Fig. 5c for suction-electrode recordings. These figures give the value of *I*_½_ from fits of response amplitudes to the Michaelis–Menten function (see Methods). The value of *I*_½_ quantifies the amount of light necessary to produce a half-maximal response. Thus the greater the value of *I*_½_, the more light required to produce a half-maximal response and the lower the sensitivity. Our recordings show that *I*_½_ was not significantly different in darkness for either whole-retina recordings (*p* = 0.357) or suction-electrode recordings (*p* = 0.675), as would be expected from the results of Fig. 5a. The value of *I*_½_ increased after both 450-nm and 560-nm bleaches, indicating a drop in sensitivity. The amplitude of this decrease in sensitivity was smaller after the 450-nm bleach compared to the 560-nm bleach by a factor of approximately two. Although we observed small changes in sensitivity with time especially for the 560-nm bleaches, these changes were not significant. We therefore averaged measurements from all the time points, which gave mean *I*_½_ values for the suction-electrode recordings of 2.6 ± 0.2 × 10^4^ after the 450-nm bleach (*n* = 75), and 5.9 ± 0.9 × 10^4^ after the 560-nm bleach (*n* = 60). This sensitivity difference was highly significant (Student’s *t*, *p* < 0.001). A similar comparison for the whole-retina recordings was also statistically significant (Student’s *t*, *p* < 0.005). Thus, with two different preparations and recording techniques, we observed higher sensitivity in mouse cones after exposure to 450-nm versus 560-nm light. Since M cones were equally sensitive to our 450-nm and 560-nm lights (Fig. 5a), the two illuminations should have produced nearly equal bleaches. The smaller decrease in sensitivity after the 450-nm bleach is consistent with a role for retinyl-lipid photoisomerization in cone pigment regeneration.

## Discussion

**Fig. 6:**
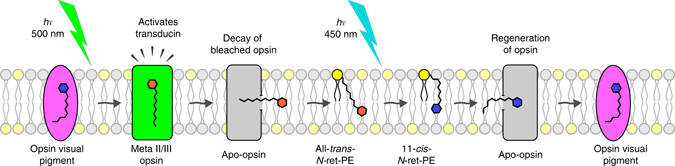
Retinyl-lipid photoregeneration of opsins in rod or cone OS. The results presented here suggest the existence of a light-driven mechanism to regenerate visual pigments in rod and cone OS through photoisomerization of retinyl-lipids. This diagram shows the phospholipid bilayer of a rod OS disk. Rhodopsin absorbs a 500-nm photon (*hv*) that photoisomerizes its 11cRAL chromophore to atRAL, converting the pigment to its active metarhodopsin II/III (meta II/III) state. After a brief signaling period, the bleached opsin decays, releasing free atRAL into the bilayer. The atRAL reversibly condenses with PE (yellow circles) to form at-*N-*ret-PE. Protonated at-*N-*ret-PE is converted specifically to 11c-*N-*ret-PE upon absorption of a 450-nm photon. Spontaneous hydrolysis of 11c-*N-*ret-PE yields free 11cRAL, which irreversibly combines with unliganded apo-opsin to form a new rhodopsin pigment. A similar process occurs in cone OS to regenerate cone opsin pigments

Rhodopsin and the cone opsin pigments require a continuous supply of visual chromophore to maintain photosensitivity in bright light. One molecule of 11cRAL is required for each photon absorbed. The endergonic conversion of atRAL to 11cRAL is carried out by multi-step enzyme pathways in RPE and Müller cells. Estimates of the maximum turnover rates suggest that the visual cycles cannot keep up with the high rates of rhodopsin and cone-opsin photoisomerization occurring in daylight^36^. Here we demonstrate a non-enzymatic mechanism for visual pigment regeneration involving photoisomerization of retinyl phospholipids in OS disk membranes (Fig. 6).

The OS membranes conduct 11cRAL and atRAL to and from the opsins during light exposure. Both retinaldehyde isomers reversibly condense with PE to form *N-*ret-PE. Protonated at-*N-*ret-PE was converted with remarkable specificity to 11c-*N-*ret-PE by blue light (Fig. 1). As an indication of this specificity, the ratio of 11cRAL to 13cRAL after exposure of at-*N-*ret-PE to 450-nm light was 37:1 (Fig. 1a). In contrast, the ratio of 11cRAL to 13cRAL at thermal equilibrium is 1:240^37^. Therefore, the 11c-isomer was enriched nearly 9000-fold by light over its equilibrium concentration during exposure to 450-nm light. This property of *N-*ret-PE^16^ allows it to serve as a source of visual chromophore. The retinoid isomerases, Rpe65 and DES1 of the canonical and non-canonical visual cycles both exhibit much lower 11c-specificity^9, 38^. The difference in free energy between atRAL and 11cRAL is 4.1 kcal/mole^37^. Both Rpe65 and DES1 use hydrolysis of a retinyl ester to drive retinoid isomerization^9, 10, 39^. The actual metabolic cost of retinoid isomerization is 7.5 kcal/mol from hydrolysis of an activated fatty acid. In contrast, the energy of a 450-nm photon is 64 kcal/mol, far more than required to convert at-*N-*ret-PE to 11c-*N-*ret-PE. Regeneration of visual chromophore through retinyl-lipid photoisomerization is a unique example of an energy-requiring metabolic reaction in mammals being powered by visible light.

The membranes of most mammalian cells contain 15–25% PE^40^, while OS disk membranes contain 38% PE^15^. The ratio of phospholipids to rhodopsin is 100:1 in OS membranes^41^, hence PE is at 38-fold molar excess over rhodopsin. This abundance of PE in OS disks promotes formation of *N-*ret-PE from free retinaldehyde released by rhodopsin, and thereby photoregeneration of visual chromophore. *N-*ret-PE is the translocated substrate for the ABCA4 flippase in OS discs^18^. ABCA4 is the product of the gene affected in recessive Stargardt macular degeneration^42^. Loss of ABCA4 in Stargardt patients and *Abca4* ^−/−^ mice causes accumulation of bisretinoids that arise through delayed clearance and secondary condensation of *N-*ret-PE with another retinaldehyde^43, 44^. We quantitated *N-*ret-PE in dark-adapted mouse retinas by comparing retinaldehydes contained in *N-*ret-PE to total retinaldehydes, which are mostly 11cRAL in rhodopsin (Supplementary Figs 2a,b and Table 1). *N-*ret-PE comprised ~7% of total retinaldehydes in a dark-adapted retina. 11cRAL not associated with rhodopsin undergoes thermal isomerization to its lower-energy isomers in the dark. This is exemplified by the higher fraction of atRAL and 13cRAL in *N-*ret-PE versus total retina following overnight dark adaptation (Table 1). Within the OS, any 11cRAL that thermally isomerizes to atRAL or 13cRAL would be quickly restored to the 11c-configuration through photoisomerization of *N-*ret-PE. Thus, 11c-*N-*ret-PE represents a protected and readily available pool of visual chromophore in photoreceptor OS.

While rods are single-photon detectors with a photoresponse that saturates in bright light, cones are less sensitive, providing color vision in bright light with a photoresponse that never saturates. Accordingly, cones seem better suited than rods to benefit from chromophore photoregeneration. We employed two experimental systems to test whether photoisomerization of *N-*ret-PE contributes to cone opsin regeneration. First, we recorded cone photovoltages from whole dorsal retinas of *Gnat1* ^−/−^ mice. We observed approximately two-fold greater sensitivity of M cones in the explants following exposure to 450-nm versus 560-nm light at the same photon flux (Fig. 5b). We also performed suction recording from isolated M-cone photoreceptors. Here again, we observed approximately two-fold greater sensitivity following a photobleach with 450-nm versus 560-nm light (Fig. 5c). These differences in cone photosensitivity are consistent with the higher levels of rhodopsin in mouse retinas following in vivo exposure to 450-nm versus 540-nm light (Fig. 4b). Hence, cones as well as rods appear to use photoisomerization of retinyl-lipids to regenerate visual pigments.

Although we used 450-nm light to uncover *N-*ret-PE photoisomerization, monochromatic light is not required for retinyl-lipid photoregeneration. In fact, white light is far more effective at photoisomerizing *N-*ret-PE than narrow-band 450-nm light, as indicated by the broad absorbance spectrum of protonated *N-*ret-PE (Supplementary Fig. 1). Given their overlapping spectra (Supplementary Fig. 1 and Fig. 4a), photoisomerization of *N-*ret-PE and bleaching of rhodopsin occur simultaneously in natural light. The rate of rhodopsin bleaching was slightly faster than the rate of *N-*ret-PE photoisomerization (Fig. 3), consistent with the observation that light exposure causes net depletion of visual pigments in retina membranes. Photoisomerization of *N-*ret-PE is therefore in the “kinetic shadow” of rhodopsin bleaching and difficult to observe.

The bleach/recovery experiment in mice (Fig. 4) allowed us to compare contributions of the enzymatic visual cycles to retinyl-lipid photoregeneration. Rhodopsin increased above its immediate post-bleach levels during the 10-min recovery period in the dark (Fig. 4b). This increase, shown by *x* in Fig. 4b, is due to the chromophore-synthesis activities of the enzymatic visual cycles. Significantly higher rhodopsin levels were seen in retinas from mice that recovered under 450-nm light, while lower rhodopsin levels were seen in retinas from mice that recovered under 540-nm light (Fig. 4b). Rhodopsin was bleached to the same extent by the 450-nm and 540-nm lights, while only 450-nm light significantly photoisomerized *N-*ret-PE (Fig. 1 and Supplementary Fig. 1). Accordingly, three factors affected rhodopsin levels in retinas from mice that recovered under 450-nm light: (i) the enzymatic visual cycles, (ii) photoisomerization of rhodopsin and (iii) photoisomerization of *N-*ret-PE; while only two factors affected rhodopsin levels in samples from mice that recovered under 540-nm light: (i) the enzymatic visual cycles and (ii) photoisomerization of rhodopsin. The contribution of *N-*ret-PE photoisomerization to rhodopsin regeneration is therefore represented by the difference in rhodopsin levels between samples that recovered under 450-nm and 540-nm light (*y* in Fig. 4b). Since *y* > *x*, photoisomerization of *N-*ret-PE contributed more to rhodopsin regeneration than did the enzymatic visual cycles under these light conditions.

The cone suction-recording experiment provided additional information about the contribution of retinyl-lipid photoisomerization to opsin pigment levels. Using the relationships in Eq. 3 and 4 (Methods), we estimated that cones exposed to 450-nm light behaved as if they contained approximately 15% more pigment than cones exposed to 560-nm light, suggesting a 15% contribution of *N-*ret-PE photoisomerization to cone opsin regeneration under these conditions. In nature, the contribution of retinyl-lipid photoisomerization to opsin pigment regeneration probably increases with intensifying ambient light. Retinyl-lipid photoregeneration may be required for sustained vision in daylight.

## Methods

### Animal use and care statement

This study was carried out in accordance with recommendations in the guide for the care and use of laboratory animals of the National Institutes of Health, and the Association for Research in Vision and Ophthalmology Statement for the use of animals in ophthalmic and vision research. The animal use protocol was approved by the University of California, Los Angeles Animal Research Committee (permit number: A3196-01). Euthanasia was performed by cervical dislocation on deeply anesthetized (xylazine 10 mg/kg and ketamine 100 mg/kg) mice. All steps were taken to minimize pain and distress in the mice.

### Synthesis and purification of at-*N*-Ret-PE

All reactions were performed under dim red light. We synthesized at-*N*-ret-PE according to published methods^45^ with modifications. Briefly, atRAL (Sigma-Aldrich) was mixed with 5.6 mg 1-oleoyl-2-hydroxy-*sn*-glycero-3-phosphoethanolamine (18:1 lyso PE, Avanti Polar Lipids) in three ml of a solution containing six volumes of methanol (Fisher Scientific), 12 volumes of chloroform (Sigma-Aldrich) and one volume of triethylamine (Sigma-Aldrich). The solution was incubated at room temperature for 1 h with gentle mixing in the dark. Three ml of one-M hydrochloric acid (EM Science) was added and the mixture was centrifuged at 3500 × *g* for 5 min. The lower organic phase (chloroform) containing at-*N*-ret-PE was collected and the upper phase was extracted again with 2 ml chloroform. The pooled extracts were further rinsed with 3 ml of one-M hydrochloric acid, and the final chloroform extracts were dried under a stream of nitrogen and dissolved in acidified methanol (20-µl trifluoroacetic acid (TFA) per liter methanol). The at-*N*-ret-PE was purified in its protonated form by reverse-phase LC (see below) with an elution time of approximately 14 min. By this method, free atRAL eluted at approximately 10 min.

### Action spectrum of at-*N*-Ret-PE

Ten µM at-*N*-ret-PE was dissolved in acidified methanol (20 µl TFA per liter) to maintain the protonated state. Its concentration was determined by absorption at 450 nm using a Shimadzu UV-2401PC UV-Vis spectrophotometer and a molar extinction coefficient of 31,300 M^−1^cm^−1^ ^15^. Triplicate samples were placed into quartz 10 mm cuvettes (SCC) covered in black paper with only the front side exposed to light. The samples were either kept in the dark or illuminated with monochromatic light at wavelengths of 325 to 650 nm with 25-nm increments at room temperature for 80 s. The monochromatic light was generated by a custom monochromator (Newport Instruments) with a xenon arc lamp. The light intensities were measured with a spectroradiometer (Black-comet CXR-SR-50, StellarNet Inc.) and adjusted (from 0.35 W/m^2^ at 325 nm to 0.18 W/m^2^ at 650 nm) such that each wavelength delivered a photon flux of 0.95 µmol photons/(m^2^ s). Two hundred µl from each sample were analyzed by normal-phase LC.

### Normal-phase LC analysis of retinoids

Retinoids were treated with 20–25 µl 5% sodium dodecyl sulfate (SDS) (for samples of at-*N-*ret-PE in methanol, SDS was not added) and 50 µl brine. To quantitate retinaldehydes in *N-*ret-PE and opsin pigments, retinaldehyde oximes were generated by addition of hydroxylamine hydrochloride (200–500 µl of 2 M solution, pH 7.0) (Sigma). The samples were mixed by vortexing and incubated at room temperature for 15 min. 2 ml of methanol were added and the samples were extracted twice with 2 ml hexane as previously described^9^. The identity of each eluted peak was established by comparing the spectra and elution times with those of authentic retinoid standards. Sample peaks were quantitated by comparing peak areas to calibration curves established with retinoid standards. Peak areas for the corresponding *syn-*oximes and *anti-*oximes were summed to quantitate each retinaldehyde isomer.

### Determination of the decay constants

Bovine rod OS were purified by published methods^46^ and used as the source of rhodopsin. Purified rod OS were dissolved in 20 mM sodium phosphate (Fisher Scientific) buffer pH 7.2 with 100 mM sodium chloride (Fisher Scientific), 2.5% sucrose (Fisher Scientific) and 2% CHAPS (Calbiochem), and diluted to an absorbance of 0.22 AU at 500 nm (the *λ*_max_ of rhodopsin). Purified protonated at-*N*-ret-PE was diluted in acidified methanol to yield an absorbance of 0.22 AU at 450 nm (the *λ*_max_ of at-*N*-ret-PE). Triplicate aliquots of purified rod OS were exposed to 500-nm monochromatic light at 0.25 W/m^2^ (1.0 µmol photons/m^2^ s) at room temperature for 0, 15, 30, 45, and 60 s. Triplicate aliquots of protonated *N-*ret-PE were exposed to 450-nm monochromatic light at 0.28 W/m^2^ (1.0 µmol photons/m^2^ s) and otherwise treated identically as the rhodopsin samples. Two hundred µl from each sample were removed and analyzed for retinoid content by normal-phase LC. The amount of rhodopsin at each time point was determined by quantitation of 11cRAL.

### Reverse-phase LC of phospholipids

Phospholipid extraction was performed with modifications to a previously published protocol^47^. All extractions were performed under dim red light. Retina homogenates from 8-week-old strain 129/Sv mice or bovine OS were extracted by the addition of 3 ml of 1:2 (vol/vol) mixture of chloroform (Sigma) and acidified methanol (Fisher) (one L methanol + 8 µl trifluroacetic acid (Sigma)) followed by brief vortexing and incubation on ice for 10 min. Next, 1.3 ml of 0.3 M NaCl were added and the samples extracted twice into one ml chloroform with centrifugation at 1750 × g for 10 min at 10 °C to separate phases. The pooled chloroform layers were transferred to 16 × 100 mm borosilicate glass test tubes and evaporated to dryness under a stream of nitrogen. Samples were dissolved in 100 µl of acidified methanol and analyzed by reverse-phase LC^48^ on an Agilent 1100 series chromatograph equipped with a photodiode-array detector using an Phenomenex Primeshere C18-HC 110 A column (250 × 4.6 mm) and a 15–0% water gradient in acidified methanol (8 µl TFA/L methanol) at a flow rate of 1.0 (gradient) to 2.4 (isocratic) ml per min. Spectra (190–550 nm) were acquired for all eluted peaks. The identity of each eluted peak was established by comparing the spectra and elution times with those of authentic retinoid and *N*-ret-PE standards. Sample peaks were quantitated by peak area.

### Mice and genotyping

All mice were reared in cyclic light. The 129/Sv wild-type control mice were purchased from Taconic Biosciences, Inc. Retinal G protein-coupled receptor knockout (*Rgr* ^−/−^) mice^29^ were generously provided by Henry Fong, genotyping protocols and strain background information were reported previously^49^. *Gnat1* ^−/−^ mice lacking rod α-transduc in^33^ were generously provided by Janis Lem. All mice were genotyped to exclude the *rd8* and *rpe65* L450M mutations. The primers for each genotyping: *rd8*, F: 5′GGTGACCAATCTGTTGACAATCC, R: 5′GCCCCATTTGCACACTGATGAC; *rpe65* codon 450, F: 5′CCTTTGAATTTCCTCAAATCAATTA, R: 5′TTCCAGAGCATCTGGTTGAG.

### Rhodopsin purification from mouse retinas

Retinas from 8-week-old wild-type (strain 129/Sv) mice were dissected under dim red light and homogenized in a glass to glass tissue grinder (Kontes) in solubilization buffer (40 mM Tris (Fisher) pH 7.2, 1% CHAPS (Fisher), and 0.1 mg/ml PMSF (Sigma)). The homogenates were spun at 17,000 × g for 15 min at 4 °C to pellet cell debris. Collected supernatants were added to 100 µl agarose beads coupled to the 1D4 antibody against rhodopsin (PureCube Rho1D4 Agarose), washed with solubilization buffer, and incubated overnight with agitation at 4 °C. Beads were combined with elution buffer (40 mM Tris pH 7.2, 1% CHAPS, 200 µM 1D4 peptide (Cube Biotech)) for 1 h at room temperature. The beads were then pelleted by centrifugation (3000 RPM for 5 min, Eppendorf 5415D centrifuge) and the rhodopsin-containing supernatants were collected. Rhodopsin was quantified spectrophotometrically at 500 nm (Shimadzu UV-2401PC UV-Vis spectrophotometer) using the molar extinction coefficient of 40,600 M^−1^cm^−1^ ^25^.

### Rhodopsin purification from bovine rod OS

Bovine rod OS were prepared from the eyes of freshly slaughtered cattle using published procedures^46^. Purified OS in 50 µl aliquots containing 2-nmoles rhodopsin were prepared in triplicate for each light condition. OS samples for rhodopsin regenerative studies were bleached on ice (12,000 lux for 45 min with a halogen lamp) to remove endogenous retinoids. The OS were supplemented with 50 µM atRAL in dimethyl sulfoxide and diluted to 200 µl with pH 6.0 phosphate-citrate buffer containing 0.1 mg/ml PMSF. Samples were incubated overnight at 4.0 °C with gentle agitation. The following day, samples were incubated at 37 °C for 30 or 60 min with gentle agitation in the dark or under 450-nm monochromatic light (20-nm bandwidth) at 0.5 W/m^2^. Samples incubated for 30 min were extracted and analyzed for retinoid content by normal-phase LC to measure production of 11cRAL. Rhodopsin was purified from samples incubated for 1 h using Rho 1D4-agarose (Cube Biotech) as described above. Absorbance spectra were acquired for all samples using a Shimadzu UV-2401PC UV-Vis spectrophotometer. Difference spectra were acquired after bleaching the samples in the same cuvette with a Novatron strobe light (3 × 1500 W). The difference spectra were normalized to the baseline (*A*_650_) and protein content (*A*_280_). Additional OS samples (dark and 450-nm light-treated) were examined for *N-*ret-PE content by reverse-phase LC, as described above. Peaks identified as *N-*ret-PE by their absorbance spectra were collected, reacted with hydroxylamine to form retinaldehyde oximes, and re-analyzed by normal-phase LC to determine the retinaldehyde content of *N-*ret-PEs.

### Photoisomerization in retina homogenates from mice

Eight to 10-week-old wild type (strain 129/Sv) and *Rgr* ^−/−^ (129/Sv background) mice were euthanized and their eyes collected. Retinas from mice of each genotype were pooled and disrupted by glass/glass homogenization (Kontes) in 6.0 ml 40 mM Tris buffer pH 7.2. The homogenates were extensively bleached (20,000 lux from a xenon arc lamp with a 400-nm UV cutoff filter for 45 min) to destroy endogenous retinoids. Protein concentrations were determined (Pierce BCA Protein Assay Kit, Thermofisher) and used for normalization of pre- and post-bleach retinoid content. Photoisomerization assays were performed on similar homogenate samples in 500 µl reactions with addition of 5% BSA, 25 µM all-*trans*-retinol, and 500 µM NADP^+^ (all from Sigma). Samples were placed in cuvettes and agitated in the dark or during exposure to 450-nm light (10-nm bandwidth with an irradiance of 0.2 W/m^2^) for 25 min at 37 °C. Retinoids were extracted from samples and analyzed by normal-phase LC, as described above.

### Blue-light dependent regeneration of rhodopsin in live mice

Triplicate sets of 8-week-old wild type (129/Sv) mice were dark adapted overnight. All mice were anesthetized as described above and their pupils dilated with one drop of 1.5% tropicamide and 2.5 % phenylephrine. Three sets of six mice were kept in the dark for the dark-adapted (DA) rhodopsin determinations (90 mice total). The remaining mice were bleached by exposure to ten 1500-W flashes of a Novatron strobe. We observed no change in the thickness of any retina layer by optical coherence tomography at one and 7 days post-exposure, indicating that the strobe light caused no retinal damage in mice. Immediately post-bleach, three sets of six mice were euthanized, their retinas collected (12 retinas per sample) and homogenized, as described above. The remaining nine sets of six mice were exposed for 10 min to one of three different light conditions: (i) darkness, (ii) 450-nm light, or (iii) 540-nm light. Both “monochromatic” light sources had a 10-nm bandwidth. The irradiances on the corneal surfaces were 0.5 W/m^2^ for the 450-nm light and 0.42 W/m^2^ for 540-nm light, to yield identical photon fluxes of 1.8 µmol photons/m^2^ s. Immediately following the 10-min recovery periods, the mice were euthanized in the dark, their retinas dissected (12 retinas per sample) and homogenized, as described above. Rhodopsin was purified from mouse retina homogenates by immunoaffinity chromatography, also as described above. After purification, rhodopsin was quantitated by difference spectra analysis. The purified rhodopsin samples were also analyzed for 11cRAL and atRAL content by normal-phase LC.

### Whole-retina and cone-suction recordings from mouse cones

*Gnat1* ^−/−^ mice (8-week-old) reared under cyclic light were dark-adapted overnight then euthanized. Their eyes were marked with a cauterization tool at the ventral pole, enucleated, and only the dorsal retina, which contains predominantly middle-wavelength sensitive (M) cones^50^, was collected. The dorsal retinas were isolated from the RPE and perfused with Ames solution (Sigma) containing an additional 1.9 g/l NaHCO_3_ and bubbled with 95% O_2_ / 5% CO_2_. For whole-retina recordings of cone responses, dorsal retinas were placed photoreceptors up on Whatman Anodisc filter membranes (Sigma) in a custom built recording chamber. Retinas were isolated and perfused with 2 mM aspartic acid and 40 μM DL-AP4 (Tocris, Bristol, UK) on the photoreceptor side and 2 mM aspartic acid and 1 mM barium chloride (Sigma) on the ganglion-cell surface. Photovoltages were measured with a DP-311 differential amplifier (Warner Instruments, Hamden, CT). For suction recordings, retinas were sliced into pieces and recordings were made from individual cone inner segments^51^. Both preparations were stimulated with an LED optical system (Cairn Research, Faversham, UK) coupled to an inverted microscope. Test flashes were delivered with a 505-nm LED through a 500-nm interference filter. Cones were bleached with either 450-nm or 560-nm illumination from a white-light LED and interference filters at 450 nm and 560 nm. The intensities of the test and bleaching lights were calibrated with a photodiode (United Detector Technology, San Diego, CA). The intensities of the 450-nm and 560-nm illuminations from the LED were nearly the same at the same diode currents, and M-cones were equally sensitive to these two wavelengths at the same diode intensities and flash durations (see Fig. 5a). Recordings were filtered with an 8-pole Bessel filter and sampled at 100 Hz. Data were displayed and analyzed with PCLAMP (Molecular Devices, Sunnyvale, CA) and Origin plotting software (OriginLabs, Cambridge, MA).

### Estimation of sensitivity

Sensitivities for Fig. 5b and c were estimated by measuring responses to two dim light intensities and to a nearly saturating bright light. The test-flash intensities were (photons μm^−2^) 2840, 9690, 200,000 before bleaches and 19,000, 43,200, 200,000 following bleaches for Fig. 5b (whole retina); and 2200, 3500, and 66,600 before bleaches and 6900, 15,500, and 66,600 after bleaches for one series of experiments, and 650, 5,410, and 95,000 before the bleaches and 9200, 40,500, and 220,000 after bleaches for a second series for Fig. 5c (suction-electrode recordings). At each intensity, 10 flashes were given at 1-s intervals for the dim flashes, or every 1.5 s for the nearly saturating flashes, and response amplitudes were averaged. The determinations at 0 min were begun 10 sec after the bleach; recovery of photoresponse and maximum response amplitude was virtually complete after 10 s, even for these large bleaches^52^. Determinations at 1–4 min were begun one to 4 min after the bleach. Each determination took a total of about 40 s. Sensitivities were then determined by fitting each series of three mean response amplitudes to the Michaelis–Menten equation (Eq. 2),

2 $$r={r}_{\rm max}\frac{I}{I+{I}_{1/2}}$$

where *r* is response amplitude in pA, *r*_max_ is the maximum value of *r*, *I* is intensity in 500-nm photons μm^−2^, and *I*_1/2_ is a constant equal to the intensity of the flash producing a half-maximal response. The Michaelis–Menten relation predicts that responses at dim light intensities (*I* <<*I*_1/2_) are directly proportional to light intensity, with a proportionality constant equal to *r*_max_/I_1/2_. This proportionality constant gives the response per unit light intensity at dim light intensities in the linear range of the Michaelis–Menten relation and is therefore equal to the sensitivity of the photoreceptor. Sensitivity is thus inversely related to *I*_1/2_, and plotting the value of *I*_1/2_ is therefore a meaningful and appropriate way to display differences in cone sensitivity provided there are no changes in *r*_max_. In Fig. 5 we therefore show the changes in the value of *I*_1/2_ after the 450-nm and 560-nm bleaches. There were no significant changes in *r*_max_.

### Estimation of cone pigment regenerated from *N-*Ret-PE

Previous experiments have shown that photoreceptor desensitization after strong bleaches is the result of two mechanisms: (i) the decrease in quantum catch produced by the decrease in concentration of unbleached pigment, and (ii) adaptation produced by activation of the cascade by bleached pigment^53, 54, 55^. These two mechanisms sum to produce a decrease in sensitivity that is well described by the equation (Eq. 3)

3 $$\frac{{S}_{F}}{S{{D}\atop{F}}}=\frac{1-F}{1+kF}$$

where *S*_*F*_ is the sensitivity of the photoreceptor, ${S{{D}\atop{F}}}$ is the sensitivity in darkness, *F* is the fraction bleached, and *k* is a constant, equal to 34–35 for mouse rods^56^ but 8.6 for salamander cones^53^.

Although no similar measurements have been made for bleached mouse cones, we can estimate the value of *k* from our data if we assume that, following the 560-nm bleach, no pigment regeneration occurred. We need first to use the values of *I*_1/2_ before and after bleaching to estimate ${S}_{F}/{S{{D}\atop{F}}}$. We observe that the Michaelis–Menten equation (Eq. 2) provides an adequate description of the responses of cones to increasing flash intensity (Fig. 5a); and that for dim flash intensities (*I*<<*I*_1/2_), the amplitude of the response (and therefore the sensitivity of the cone) is inversely proportional to *I*_1/2_. Therefore, we can estimate ${S{{D}\atop{F}}}$ as 2560/59,000 or 0.043 after the 560-nm bleach, and 2410/26,000 or 0.093 after the 450-nm bleach.

Now, if no pigment regeneration had occurred after the 560-nm bleach, *F* = 0.85 and we can solve equation in Eq. 3 for *k* and obtain a value of 2.9, considerably lower than for salamander cones. We now insert this value into Eq. 3 and solve for the fraction bleached (Eq. 4).

4 $$F=\frac{1-\frac{{S}_{F}}{S{{D}\atop{F}}}}{1+k\frac{{S}_{F}}{S{{D}\atop{F}}}}$$

If *k* is 2.9 and, after the 450-nm bleach, ${S}_{F}/{S{{D}\atop{F}}}$ is 0.093, then *F* is 0.71 instead of 0.85. The cones are behaving as if the 450-nm light had produced approximately 15% less bleached pigment than the 560-nm bleach, presumably because of retinyl-lipid photoisomerization. We note that if we have underestimated the value of *k*, and it is in fact larger and nearer to that for salamander cones or mouse rods, the value of *F* from Eq. 4 would have been smaller and the amount of pigment regenerated through *N-*ret-PE photoisomerization even larger.

### Reproducibility and statistical analysis of mouse studies

No power studies were performed on mouse experiments. No experimental animals were excluded from analysis. No randomization or blinding was used in the mouse studies. Statistical significance (*p*-values) were determined either with a two-tailed Welch’s *t*-test or Student's *t*. *p*-values of less than 0.05 were considered significant.

### Data availability

All data generated and analyzed during this study are included in this published article and its Supplementary Information files, and available from the corresponding author upon request.

## Electronic supplementary material

Supplementary Information Supplementary Figures
