# Supplementary material for: Blue light regenerates functional visual pigments in mammals through a retinyl-phospholipid intermediate
Source: Nat Commun. 2017 May 4;8:16. doi: 10.1038/s41467-017-00018-4 (PMC5432035; doi:10.1038/s41467-017-00018-4)
Supplement: Supplementary file 1 — Supplementary Figures [file 41467_2017_18_MOESM1_ESM.pdf]

SUPPLEMENTARY INFORMATION

SUPPLEMENTARY FIGURES

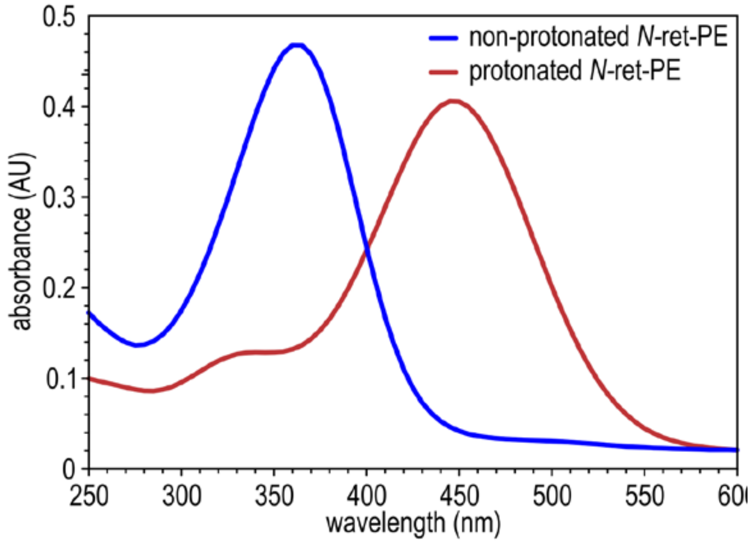

Supplementary Figure 1. Absorbance spectra of protonated and non-protonated *N*-ret-PE. Absorbance spectra for synthetic *N*-ret-PE were acquired in acidified (red curve) or alkalinized (blue curve) methanol. The λ_{max} is 450 nm for protonated- and 365 nm for non-protonated-*N*-ret-PE.

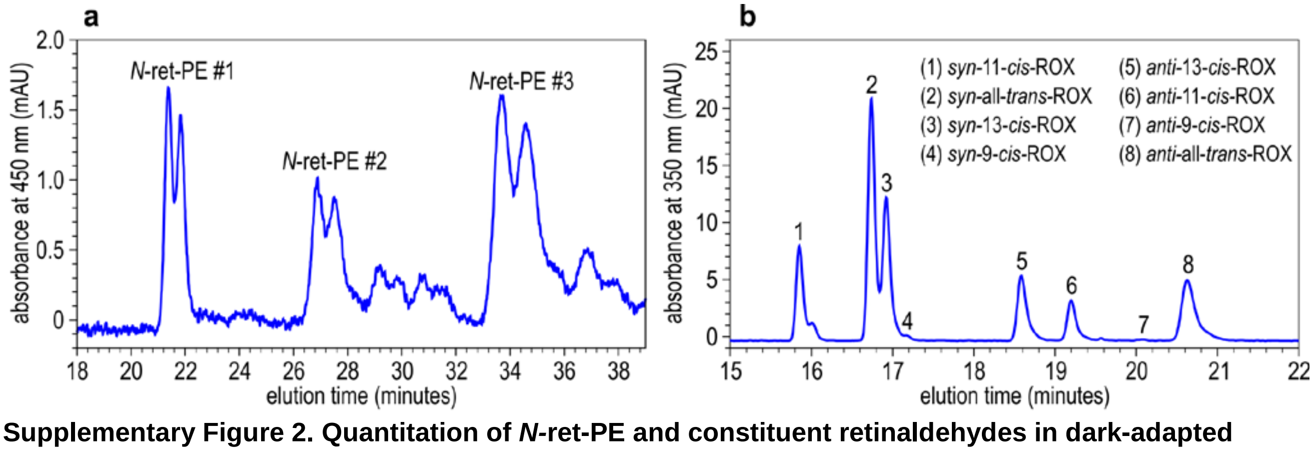

Supplementary Figure 2. Quantitation of *N*-ret-PE and constituent retinaldehydes in dark-adapted mouse retinas. (a) Reverse-phase chromatogram. Phospholipids were extracted from dark-adapted mouse retinas and separated by reverse-phase LC. An excerpt of the chromatogram is shown here. Three doublet peaks were identified with λ_{max} = 450 nm (*N*-ret-PE #1, *N*-ret-PE #2 and *N*-ret-PE #3), representing different fatty-acyl forms. Fractions containing these peaks were collected and pooled. **(b)** Normal-phase chromatogram. The pooled *N*-ret-PE sample was reacted with hydroxylamine to form retinaldehyde oximes, which were separated by normal-phase LC. An excerpt of the chromatogram containing the retinaldehyde oximes is shown. The *syn*- and *anti*-oximes of the four retinaldehyde isomers found are identified in the inset. Retinaldehyde levels are shown in Table 1.

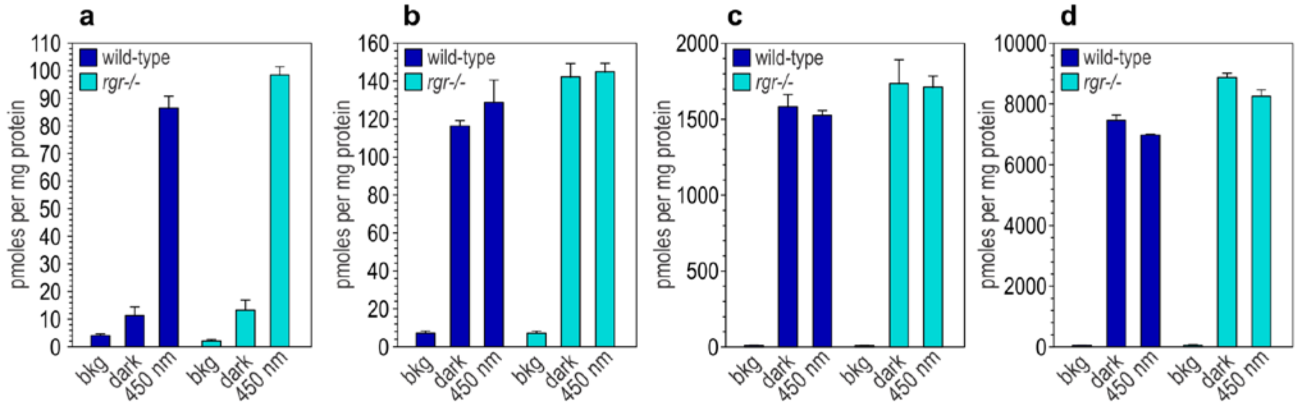

Supplementary Figure 3. Blue-light dependent synthesis of 11cRAL by wild type and *Rgr-/-* mouse retinas. Homogenates of retinas from wild type and *Rgr-/-* mice were photobleached to remove endogenous retinoids and incubated with atROL in the dark or during exposure to 450-nm light. Homogenates then were extracted into hexane and analyzed by normal phase LC. The retinaldehyde isomers: **(A)** 11cRAL, **(B)** 9cRAL, **(C)** 13cRAL, and **(D)** atRAL are shown as pmoles per mg protein in retinas immediately post-bleach (bkg), following incubation in the dark (dark), and following incubation in 450-nm light (450 nm). The dark and 450-nm light exposed samples were incubated with at-retinol. Error bars show mean ± S.D. (n=4). Note the approximately eight-fold higher 11cRAL in wild type and *Rgr-/-* retina homogenates incubated in 450-nm light versus darkness. Also note the similar levels of 11cRAL in wild type and *Rgr-/-* homogenates incubated in 450-nm light.
